# Primary and Chronic HIV Infection Differently Modulates Mucosal Vδ1 and Vδ2 T-Cells Differentiation Profile and Effector Functions

**DOI:** 10.1371/journal.pone.0129771

**Published:** 2015-06-18

**Authors:** Eleonora Cimini, Chiara Agrati, Gianpiero D’Offizi, Chrysoula Vlassi, Rita Casetti, Alessandra Sacchi, Raffaella Lionetti, Veronica Bordoni, Nicola Tumino, Paola Scognamiglio, Federico Martini

**Affiliations:** 1 Cellular Immunology Laboratory, National Institute for Infectious Diseases “Lazzaro Spallanzani” I.R.C.C.S., Via Portuense, 292, 00149, Rome, Italy; 2 Virology Laboratory, National Institute for Infectious Diseases “Lazzaro Spallanzani” I.R.C.C.S., Via Portuense, 292, 00149, Rome, Italy; 3 Clinical Department, National Institute for Infectious Diseases “Lazzaro Spallanzani” I.R.C.C.S., Via Portuense, 292, 00149, Rome, Italy; 4 Gastrointestinal Endoscopy Unit, National Institute for Infectious Diseases “Lazzaro Spallanzani” I.R.C.C.S., Via Portuense, 292, 00149, Rome, Italy; 5 Epidemiology Unit, National Institute for Infectious Diseases “Lazzaro Spallanzani” I.R.C.C.S., Via Portuense, 292, 00149, Rome, Italy; Cardiff University School of Medicine, UNITED KINGDOM

## Abstract

Gut-associated immune system has been identified as a major battlefield during the early phases of HIV infection. γδ T-cells, deeply affected in number and function after HIV infection, are able to act as a first line of defence against invading pathogens by producing antiviral soluble factors and by killing infected cells. Despite the relevant role in mucosal immunity, few data are available on gut-associated γδ T-cells during HIV infection. Aim of this work was to evaluate how primary (P-HIV) and chronic (C-HIV) HIV infection affects differentiation profile and functionality of circulating and gut-associated Vδ1 and Vδ2 T-cells. In particular, circulating and mucosal cells were isolated from respectively whole blood and residual gut samples from HIV-infected subjects with primary and chronic infection and from healthy donors (HD). Differentiation profile and functionality were analyzed by multiparametric flow cytometry. P-HIV and C-HIV were characterized by an increase in the frequency of effector Vδ1-T cells both in circulating and mucosal compartments. Moreover, during P-HIV mucosal Vδ1 T-cells expressed high levels of CD107a, suggesting a good effector cytotoxic capability of these cells in the early phase of infection that was lost in C-HIV. P-HIV induced an increase in circulating effector Vδ2 T-cells in comparison to C-HIV and HD. Notably, P-HIV as well as HD were characterized by the ability of mucosal Vδ2 T-cells to spontaneously produce IFN-γ that was lost in C-HIV. Altogether, our data showed for the first time a functional capability of mucosal Vδ1 and Vδ2 T-cells during P-HIV that was lost in C-HIV, suggesting exhaustion mechanisms induced by persistent stimulation.

## Introduction

A hallmark of HIV infection is the early, dramatic and irreversible impairment of mucosal CD4 T-cells, particularly in gut lymphoid tissue enclaves [1,2]. The massive loss of mucosal memory CD4 T-cells persists during infection course, with little or no repopulation even after long-lasting combined antiretroviral treatment (cART) [3]. Moreover, CD4 T-cell reduction is associated to dramatic alterations of the mucosal microenvironment, causing intestinal dysfunction and malabsorption, loss of epithelial barrier integrity, and severe enteropathy, and amplifying the inflammatory response [4,5]. Translocation of microbial products from the gut, in turn, correlates with increased immune activation in chronic HIV infection, and may further damage the immune system by increasing viral and activation-induced T-cell death, by reducing T-cell reconstitution and functionality [6].

The innate mucosal immune system represents a key sentinel acting in the early phase of infections by inhibiting microbial replication and by orchestrating the subsequent adaptive immune response. In this context, the ability of γδ T-cells to respond to stress-antigens or phosphoantigens [7] highlights their possible key role in fighting invading pathogens through broad antiviral mechanisms [8]. However, very limited data are available on human mucosal γδ T-cells during HIV infection [9,10]. Among γδ T-cells, there are two main subsets, expressing either the first variable region (Vδ1) or the second variable region (Vδ2) of the delta locus for T-cell receptor (TCR) [11]. In healthy subjects (HD), Vδ1 T-cells are found predominately at mucosal sites, and are known to respond to non-classical MHC molecules expressed on stressed cells [7]. In contrast, Vδ2 T-cells represent among 70% of circulating γδ T-cell subset, and are able to respond to phosphoantigens without MHC restriction [12]. Many experimental evidences suggest a direct role of circulating Vδ2 T-cells during HIV disease. They may exert a direct anti-HIV role by secreting chemokines competing for HIV entry co-receptors as well as other soluble antiviral factors, and by killing infected cells by cytotoxic natural killer-like mechanisms [13]. During HIV infection, circulating γδ T-cells are deeply affected, and the balance between Vδ1 and Vδ2 T-cells is disrupted [14]. Indeed, an increase of Vδ1 T-cells [15] and a parallel a dramatic loss of Vδ2 T-cells was observed in the peripheral blood of HIV patients [14,16]. Finally, a persistent functional impairment of Vδ2 T-cells was observed in chronically HIV-infected patients, probably due to the induction of cellular exhaustion or anergy [17–19].

Human mucosal T-cells are mainly T-cell receptor αβ+CD8+ in the small intestine, and only a small fraction (about 15%) usually express TCR γδ [20]. In literature, an increase of mucosal γδ T lymphocytes was observed in celiac disease [21], in cutaneous pathologies (dermatitis herpetiformis) [22], in cutaneous leishmaniasis [23], in tuberculous lymphadenitis [24] and leprosy [25]. Interestingly, Nilssen et al. demonstrated that mucosal γδ T-cells were increased in chronic HIV-infected patients independently from cART [9,26]. However, very limited data are available on differentiation and activation profile and effector functions of human mucosal γδ T-cells during HIV infection.

Aim of this work was to evaluate how primary and chronic HIV infection may differently affect phenotype and function of circulating and mucosal Vδ1 and Vδ2 T-cells.

## Materials and Methods

### Ethics statement

The study was approved by the local Ethical Committee (approval number: 49/2009) and all participants gave written informed consent.

### Enrolled subjects

Primary-HIV infected patients (P-HIV, n = 15), chronic-HIV infected patients (C-HIV, n = 14) and healthy donors (HD, n = 35) were enrolled at the INMI “L. Spallanzani”. Primary HIV infection was defined by a negative or indeterminate HIV-1 Western blot with simultaneous positive plasma HIV-RNA. Patients were defined as chronically infected if they were diagnosed with HIV at least 12 months before the enrolment in this study.

Clinical features were reported in Table 1. 3/14 of C-HIV were under cART treatment. 1/15 of P-HIV and 3/14 of C-HIV presented HCV or HBV co-infections. However, no differences in the parameters analysed in this study were observed between treated and untreated subjects and between mono-infected and co-infected HIV subjects (data not shown). Blood and duodenal samples from P-HIV were collected before starting cART.

**Table 1:** Clinical features of enrolled subjects.

	HD	Primary HIV	Chronic HIV
**Subjects**	35	15	14
**Gender (F/M)**	20/15	2/13	0/14
**Age** (mean ± SD)	48,9±8,3	38,7±10,5	47±12,2
**CD4/μl** (mean ± SD)	NT	547.7±426.6	386.1±187.7
**Viral Load**			
NT	35	0	0
undetected	0	0	3
1000–100.000 cp/ml	0	3	0
100.000-2x10^6^cp/ml	0	12	11
**Therapy**	0/35	0/15	3/14 *
**Biopsy**	13	15	14
**Co-infections**			
HBV	NT	1/15	0
HCV	NT	0	3/14

HD, healthy donors; NT, not tested;

* HIV therapy: pt1: lamivudine, zidovudine, nevirapine; pt2: atazanavir, ritonavir, tenofovir, emtricitabine; pt3: ritonavir, darunavir, tenofovir, emtricitabina.

### Peripheral Blood and Duodenal Samples

Peripheral blood samples were collected from 15 P-HIV and 14 C-HIV and 35 HD. Residual duodenal samples were obtained from the same 15 P-HIV, 14 C-HIV and from 13/35 of the same HD in the day of biopsy. HIV infected subjects and Healthy donor did not significantly differ for gender and age. All subjects (P-HIV, C-HIV, HD) performed upper gastrointestinal endoscopy for several conditions: suspect of gastroesophageal reflux disease, gastritis, nausea or pain. All duodenal biopsies resulted negative for H. Pylori infection.

### Isolation of peripheral blood mononuclear cells (PBMC) and gut cells

PBMC were isolated by density gradient centrifugation (Lympholyte, Cedarlane, Canada). Mucosal cells were isolated by digesting duodenal biopsies with enzymatic mix of Collagenase (10mg/ml, Sigma), Hyaluronidase (1mg/ml, Sigma) and DNAse (200mg/ml, Sigma) in HBSS (Hank balance salt solution) buffer for 2 hour at 37°C by Gentle MACS Dissociator (Milteny Biotec). After digestion, cellular suspension was filtered and washed once with complete medium (RPMI with 10% Fetal Bovine Serum, 2mM L-glutamine, 2 mM/ml Penicillin and 50 μg/ml Streptomycin, EuroClone, Italy).

### Antibodies and flow cytometry

Phenotype analysis of Vδ1 and Vδ2 T-cells was performed by using the following monoclonal antibodies: anti-Vδ2 FITC (clone IMMU389, Beckman Coulter Immunotech, Marseille, FR), anti-Vδ1 FITC (clone TS8.2; Thermo Scientific, USA), anti-CD3 PerCP (clone SK7, BD Pharmingen, San Jose, CA, USA), anti-CD27 APC (clone L128, BD Biosciences, San Jose, CA, USA), anti-CD45RA CY-Chrome (clone HI100, BD Biosciences San Jose, CA, USA), anti-CD3 AMCyan (clone SK7, BD Biosciences, Usa). The differentiation profile of Vδ1 and Vδ2 T-cells was analysed by monitoring the expression of CD45RA and CD27 markers. Specifically, Naïve was defined as CD45RA+CD27+, Central Memory as CD45RA-CD27+, Effector Memory as CD45RA-CD27-, and terminally differentiated as CD45RA+CD27-. Briefly, PBMC (1×10^6^ cells/ml) were incubated with mAbs cocktail for 10 min a 4°C, washed once and fixed with 1% paraformaldehyde (1% PFA, Sigma, St. Louis, MS). Sample acquisition and data analysis were performed by a FACS Canto II Flow Cytometer (Becton Dickinson) by using Diva software.

### Cytotoxicity/Cytokines production

To analyze cytotoxicity/cytokines production by γδ T-cells, PBMC/Mucosal cells from P-HIV, C-HIV and HD were cultured for 18 hours in complete medium (spontaneous release) or with PMA/Iomomycin, as positive control (PMA: 50nM, Ionomycin: 1μM, Sigma Aldrich) in the presence of Brefeldin A (10 μg/ml, Serva, Germany) to block cytokines secretion. Moreover, for the analysis of Vδ1 T-cells, CD107a PE (clone H4A3, BD Pharmingen, Usa) was added in culture. Surface staining was performed by staining cells for 15 minutes at 4°C with two different surface antibodies-cocktails: I) anti-Vδ2/-FITC/CD3 AM-Cyan; II) anti-Vδ1 FITC/CD3 AM-Cyan. After incubation, cells were washed once (PBS 1X, 0.1% NaN3, 1% BSA), fixed with 1% PFA (Sigma, St. Louis, MS) and stained in permeabilizing solution (PBS 1X, 0.1% NaN3, 1% BSA, 0.5% saponin) at room temperature with an APC-labeled IFN-γ-specific antibody (clone B27, BD Pharmingen, Usa). After washing (PBS 1X, 0.1% NaN3, 1% BSA, 0.1% saponin), cells were acquired by flow cytometry (FACS Canto II, Becton Dickinson) and data were analyzed by using Diva software.

### Statistical analysis

Statistical significance was determined by GraphPad Prism software. Differences in the median values among groups were evaluated by non parametric Mann-Whitney test and a p–value <0.05 was accepted as statistically significant.

## Results

### Primary and chronic HIV infection differently modulates mucosal Vδ1 and Vδ2 T-cells frequency and differentiation profile

To study whether primary (P-HIV) and chronic (C-HIV) HIV infection may affect circulating and mucosal γδ T-cells, the frequency and differentiation profile of Vδ1 and Vδ2 T-cells were analysed in HD, P-HIV and C-HIV in two different compartments: i) peripheral blood and ii) gut tissue.

In HD, Vδ1/Vδ2 T-cell ratio was inverted in the mucosal compartment when compared to peripheral blood (mucosal Vδ1/Vδ2: 13.30 vs. circulating Vδ1/Vδ2: 0.33, p<0.0001; data not shown), confirming a preferential localization of Vδ1 T-cells in gut tissue [7,27].

**Fig 1:**
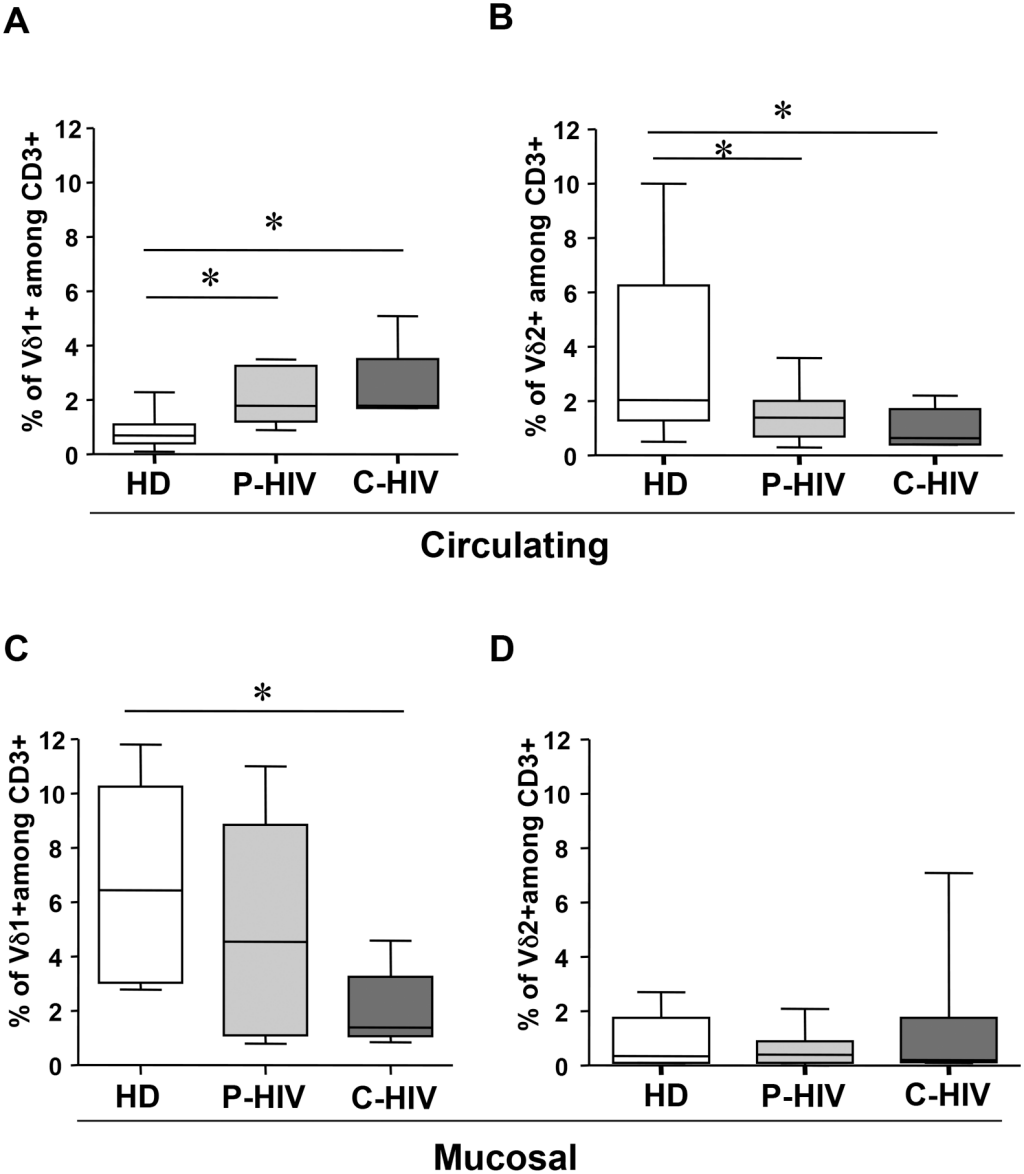
Vδ1 and Vδ2 T-cells in peripheral and mucosal compartments in HIV-patients and HD. Vδ1 and Vδ2 T-cells frequency was analyzed by flow cytometry in peripheral (Panels A-B) and in mucosal (Panels C-D) compartments of 15 P-HIV, 14 C-HIV patients and 35 HD. Data were considered significant with a p<0.05. Center line represents median; box represents interquartile range (IQR); whiskers represent range.

In the circulating compartment (Fig 1, Panels A-B), P-HIV and C-HIV were associated to a significant increase of Vδ1 T-cells (p<0.006, Fig 1, Panel A) and to a parallel decrease of Vδ2 T-cells (p<0.0034, Fig 1, Panel B) when compared to HD.

In the mucosal compartment (Fig 1, Panels C-D), the frequency of Vδ1 T-cells was similar in P-HIV and in HD. In contrast, C-HIV was associated to a significant decrease of Vδ1 T-cells (p<0.03, Fig 1, Panel C) in comparison to HD. Interestingly, in the gut tissue, the frequency of Vδ2 T-cells was not affected by HIV infection (Fig 1, Panel D).

**Fig 2:**
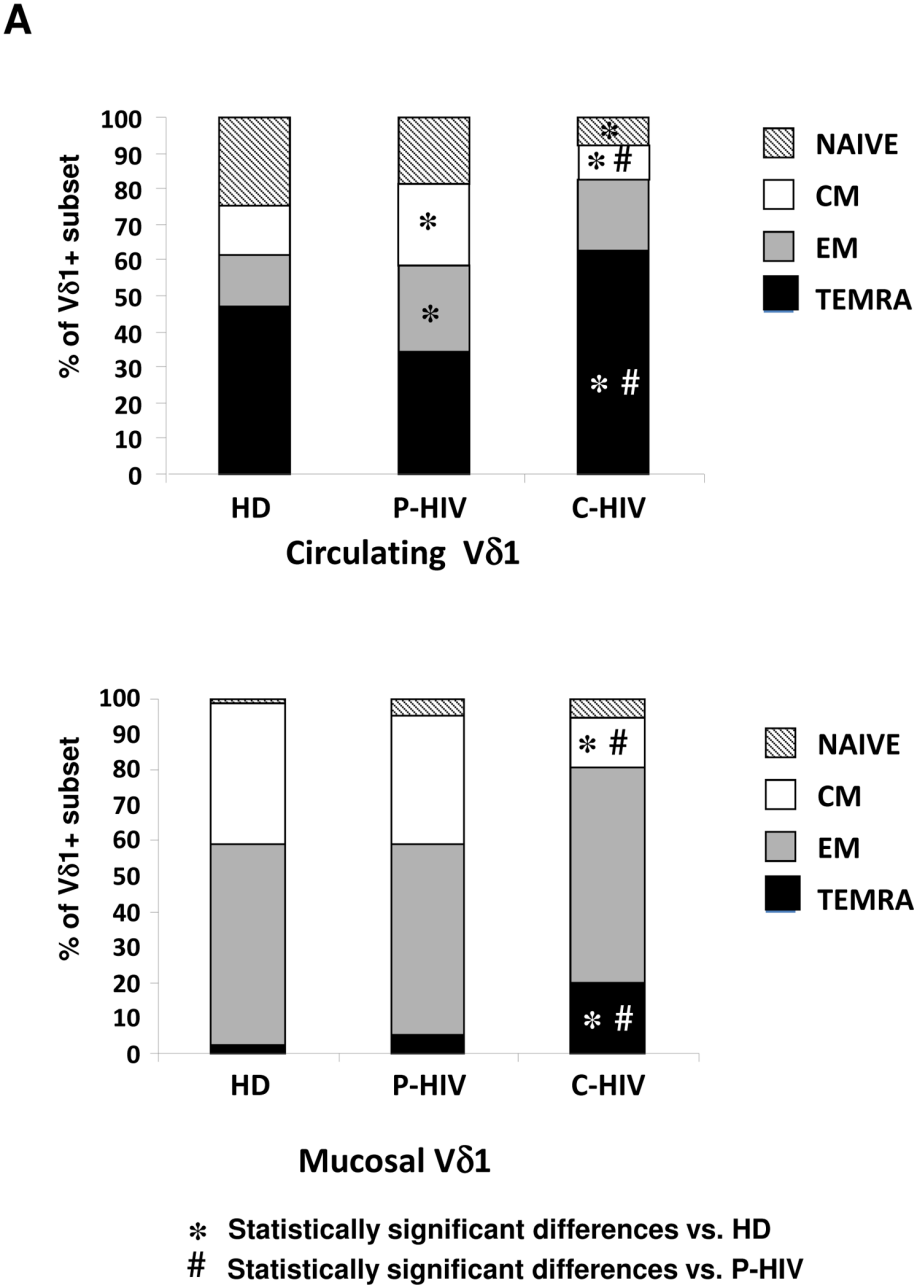
Vδ1 T-cells differentiation in peripheral and mucosal compartments from HIV-patients and HD. The frequency of Naïve (CD45RA+CD27+, hatched bars), Central Memory (CD45RA-CD27+, white bars), Effector Memory (CD45RA-CD27-, grey bars) and Terminally differentiated (CD45RA+CD27-, black bars) Vδ1 T-cells cells was analysed in peripheral (Panel A) and mucosal (Panel B) compartments in 15 P-HIV, 14 C-HIV patients and 35 HD by flow cytometry. Data were considered significant with a P<0.05.

The analysis of differentiation profile of circulating Vδ1 T-cells (Fig 2, Panel A) showed that P-HIV was associated to a significant increase of Central Memory (CM) Vδ1 T-cells (p<0.028), and Effector Memory (EM) Vδ1 T-cells (p<0.016) in comparison to HD. Moreover, in C-HIV a decrease of Naïve Vδ1 T-cells (p<0.04) and CM Vδ1 T-cells (p<0.028) were shown, paralleled by an increase in Terminal Effector Memory (TEMRA) Vδ1 T-cells (p<0.029) when compared to HD. Finally, when comparing P-HIV vs. C-HIV infection, an advanced differentiation profile was associated to C-HIV, with a significant increase of TEMRA Vδ1 T-cells (p<0.02), probably due to a persistent antigenic stimulation.

In the mucosal compartment (Fig 2, Panel B), the differentiation profile of Vδ1 T-cells was similar in P-HIV and in HD. In contrast, C-HIV was associated to a significant decrease of CM Vδ1 T-cells (p<0.035), paralleled by a significant increase of TEMRA cells (p<0.04) when compared both to P-HIV and HD.

**Fig 3:**
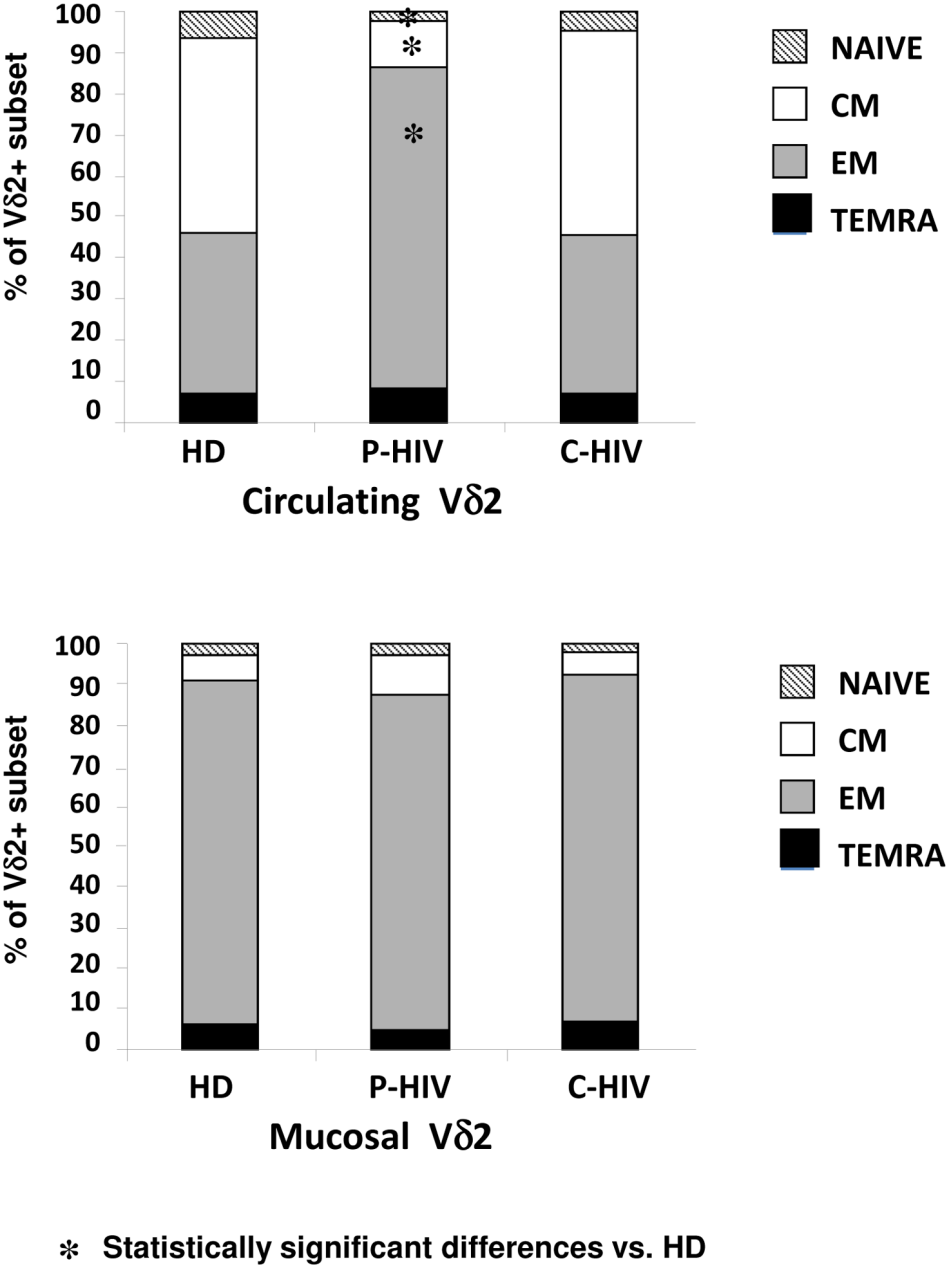
Vδ2 T-cells differentiation in peripheral and mucosal compartments from HIV-patients and HD. The frequency of Naïve (CD45RA+CD27+, hatched bars), Central Memory (CD45RA-CD27+, white bars), Effector Memory (CD45RA-CD27-, grey bars) and Terminally differentiated (CD45RA+CD27-, black bars) Vδ2 T-cells cells was analysed in peripheral (Panel A) and mucosal (Panel B) compartments in 15 P-HIV, 14 C-HIV patients and 35 HD by flow cytometry. Data were considered significant with a P<0.05.

P-HIV was associated to a significant decrease of Naïve Vδ2 T-cells (p<0.03) and CM Vδ2 T-cells (p<0.0002), and a corresponding significant increase of EM Vδ2 T-cells (p<0.0003). Circulating Vδ2 T-cells showed a similar differentiation profile during C-HIV as observed in HD (Fig 3, Panel A). Notably, in the mucosal compartment both P-HIV and C-HIV did not induce any significant differences in Vδ2 T-cells phenotype (Fig 3, Panel B).

### HIV infection interferes with mucosal Vδ1 and Vδ2 T-cell functions

Vδ1 and Vδ2 T-cell function was analysed by evaluating Vδ1 CD107a expression and Vδ2 IFN-γ production. Immunological competence of Vδ1 and Vδ2 T-cells was verified by a positive response to mytogenic stimulation (data not shown).

**Fig 4:**
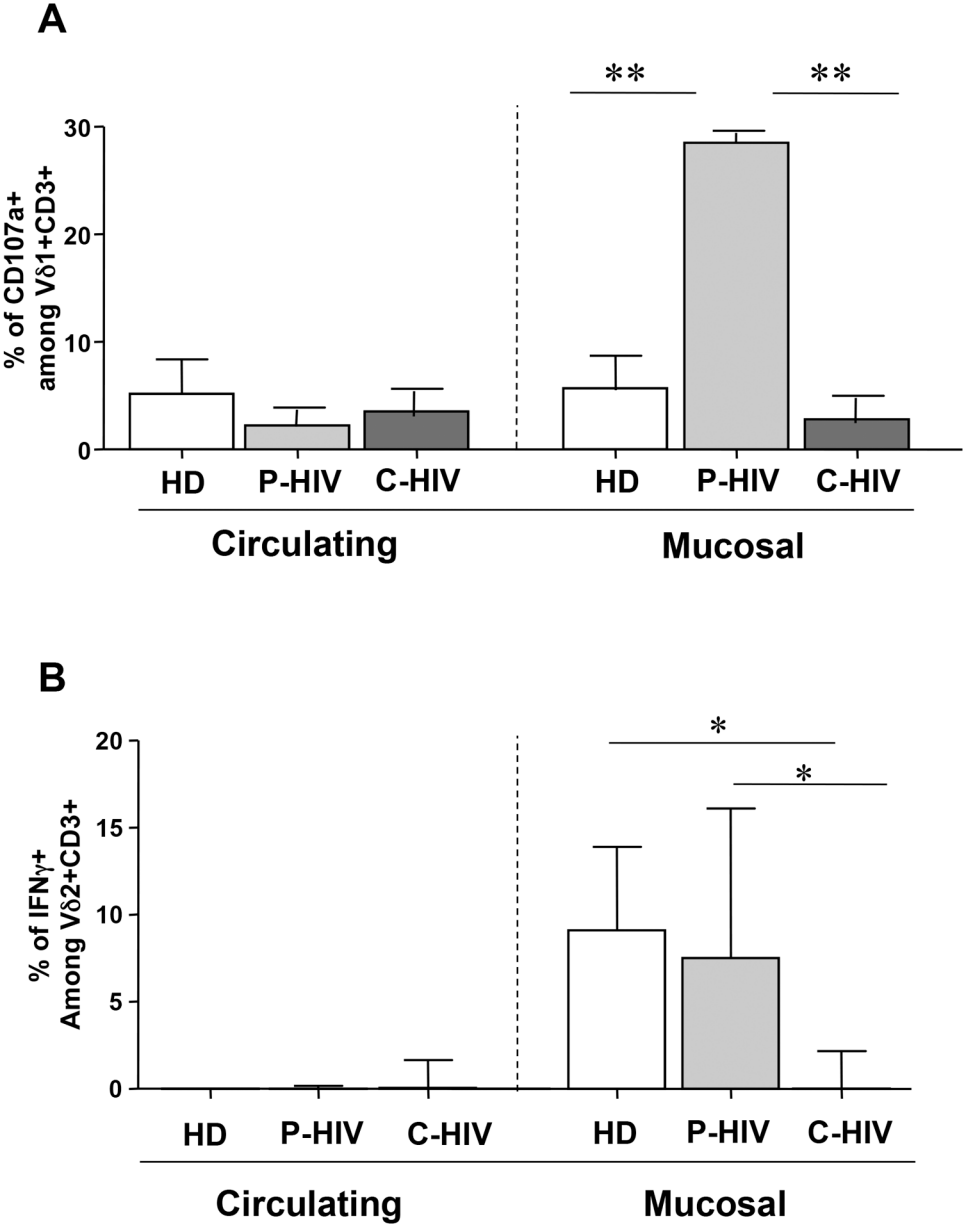
HIV infection modulates Vδ1 and Vδ2 T-cells functionality. CD107a expression was analyzed in peripheral and in mucosal compartments in 3 P-HIV, 3 C-HIV patients and 3 HD by flow cytometry (Panel A). Basal IFNγ production by Vδ2 T-cells was analyzed in peripheral and in mucosal compartments in 4 P-HIV, 4 C-HIV patients and 4 HD by flow cytometry (Panel B). Data were considered significant with a P<0.05.

The analysis of cytotoxic capability of peripheral and mucosal Vδ1 T-cells was performed by monitoring spontaneous CD107a expression by flow cytometry (Fig 4, Panel A). Independently from HIV infection, a similar frequency of CD107a+ Vδ1 T-cells was observed in circulating compartment (Fig 4, Panel A). In contrast, in the mucosal compartment, P-HIV was characterized by a higher level of CD107a+ Vδ1 T-cells when compared to both C-HIV and HD (p<0.009 for both comparisons).

The functional capability of Vδ2 T-cells was analyzed by monitoring IFN-γ production *ex vivo* by flow cytometry (Fig 4, Panel B). Independently from HIV infection, no IFN-γ-producing Vδ2 T-cells were observed in circulating compartment (Fig 4, Panel B). In contrast, in the mucosal district, we found a significant frequency of Vδ2 T-cells producing IFN-γ both in HD and in P-HIV, suggesting a good effector capability. Notably, this ability was lost during C-HIV (p<0.05 for both comparison), probably due to exhaustion mechanisms.

## Discussion

Gut-associated immune system has been identified as a mayor battlefield during the early phases of HIV infection [1,3], and the damages inflicted on the mucosal barrier integrity are considered as a relevant pathogenetic mechanism, by inducing a huge expansion in the number of HIV-susceptible cells [4,5,28].

Among mucosal immune cells, γδ T-cells are known to be able to perform an antiviral activity by exerting a cytolytic activity, by releasing antiviral soluble factors [29,30] and by orchestrating the deployment of specific immune response [31,32]. Moreover, γδ T-cells are known to be specifically impaired during HIV infection [13,14,17,19]. Despite the relevant role in mucosal immunity, few data are available on gut-associated γδ T-cells during HIV infection [9,10]. In particular, Nilssen & Brandtzaeg found an increase of total γδ T-cells, but not distinguishing Vδ1 and Vδ2 T-cells [9]; for this reason their results cannot be compared to those showed in the present paper. Aim of this work was to evaluate how primary and chronic HIV infection differently affects number, differentiation profile and response capability of Vδ1 and Vδ2 T-cells in the mucosal compartment.

We showed that chronic HIV infection is associated to a decrease of mucosal Vδ1 T-cells and a parallel increase of circulating Vδ1 T-cells. This effect was early shown as a trend during primary infection and become significant during the chronic phase of HIV infection, and was not modified in those patients under antiretroviral treatment, thus confirming previous data [9]. Several possible mechanisms could explain this finding such as the migration of Vδ1 T-cells from gut to peripheral blood, driven by chemokine receptors modulations [33], or the selective depletion of mucosal Vδ1 T-cells in gut, as observed for other cellular subtypes such as CD4 T-cells [34]. Moreover, HIV infection is able to modulate mucosal Vδ1 T-cell differentiation with an early expansion of memory and effector cells in primary infection, and a subsequent expansion of TEMRA cells during the chronic phase. Notably, in the primary phase of infection mucosal Vδ1 T-cells acquired a cytotoxic capability that was lost in the chronic phase, suggesting a functional impairment of Vδ1 T-cells due to a persistent stimulation, as observed in other clinical conditions such as gastric cancer [35].

In the circulating compartment, P-HIV drives an accumulation of effector-memory Vδ2 T-cells which later resolves in chronic infection, suggesting an attempt of Vδ2 T-cells to fight the new infection during P-HIV. The immunological failure in C-HIV was indeed characterized by a constriction of effector Vδ2 T-cells. In the mucosal compartment, HIV infection was not found to induce any change in Vδ2 T-cells frequency and differentiation. Nevertheless, mucosal Vδ2 T-cells were found able to spontaneously produce IFN-γ in HD as well as in primary HIV infection, but this capability was lost in chronic HIV, suggesting a functional exhaustion mechanism during the chronic phase of HIV infection, similarly to other mucosal cells such as CD8 T-cells [36], CD4 T-cells [34], NK cells [37], B cells [38], and Dendritic cells [39]. The analysis of exhaustion markers such as PD-1, Tim-3, CTLA4, CD95 on γδ T-cells in mucosal tissue is mandatory in order to depict the anergy mechanisms induce by infection. Moreover, the analysis of mucosal γδ response to specific stimulation, not allowed in the present paper due to the low cell counts, should be very interesting, and is among the main points of an ongoing project.

Our data shows that HIV infection is able to differently modulate γδ T-cells in the blood and in the gut mucosa. Circulating Vδ1 and Vδ2 T-cells seem to be strongly affected by P-HIV and C-HIV in the frequency and differentiation profile, but these modifications do not seem to have any impact on their spontaneous function capability. It would be very interesting to verify possible modulation in their effectiveness in responding to specific stimulation during P-HIV and C-HIV. These differences may be accounted to different tissue-specific signals or to different Vδ1 and Vδ2 subsets in the gut and in the blood. Peripheral blood and gut mucosa may differ for strength or the duration of the stimulation, for co-stimulation profile, for dendritic cells effectiveness and for cytokine milieu, thus resulting in different impact on γδ T cell effectiveness. Alternatively, Vδ1 and Vδ2 populations in the two compartments may express different γ—chains, presenting distinct sensitivity and effector functions. A specific depletion of circulating Vγ9JP+ Vδ2 T-cells has been described in chronic HIV patients [40], and was associated to the exhaustion of response to their antigens. In this context, the analysis of Vγ chain usage in gut mucosa may help to clarify possible tissue-specific compartmentalization.

In conclusion, we showed for the first time that mucosal Vδ1 and Vδ2 T-cells are differently modulated in phenotype and function during primary and chronic HIV infection. In particular during the primary phase of HIV infection an *ex vivo* functional response of both Vδ1 and Vδ2 T-cells was observed, suggesting a role of γδ T-cells in the mucosal early antiviral response. In contrast, the functional impairment of both mucosal Vδ1 and Vδ2 T-cells observed in chronic HIV infection suggests exhaustion mechanisms induced by persistent stimulation. Work is in progress to evaluate how an early antiviral treatment could enhance the effector capability of this immune compartment, possibly resulting in an improved clinical outcome.
